# Response to: Comment on “Invisible Victims: Delayed Onset Depression among Adults with Same-Sex Parents”

**DOI:** 10.1155/2016/6834618

**Published:** 2016-12-19

**Authors:** D. Paul Sullins

**Affiliations:** The Catholic University of America, Washington, DC, USA

 I appreciate the opportunity to respond to Dr. Frank's letter [1] about my article [2] and applaud Hindawi fostering a free and open exchange. Frank's complaint that I “fudged” the sample to bias the results in ways that are “damning” to gay and lesbian parents is emphatically false. Frank's claims are based on multiple confusions and errors, mischaracterize the state of knowledge, and use special pleading. To the extent some of his points have merit they tend to undermine not my study but rather others showing benign findings for children with same-sex parents and suggest I have if anything understated the level of harm for such children.

*No Harm Studies: 74, or Fewer than 10?* Frank characterizes my findings as an “outlier” from 74 studies collected on his website showing no disadvantage for children of gay or lesbian parents. But there are many other studies he did not select, which report difficulties in same-sex partnerships similar to my study. I cited three such studies concerning health difficulties and intimate partner violence (IPV). Messinger's conclusion, for example, is very similar to mine: “concerns over ‘airing the dirty laundry' of an already stigmatized community alongside researcher prejudice or indifference cannot justify treating GLB [Gay, Lesbian, Bisexual] IPV victims as invisible, leaving them without support in a painful and potentially dangerous environment.” [3] My study is not an outlier but is in line with the concerns and approach of these other studies.

Frank also does not mention that his website also includes four studies that* do* show disadvantage for children of gay or lesbian parents. Three of these studies employ three separate large population samples, finding similar levels of disadvantage [4–6]. By contrast, the 74 studies include only two or three which use population samples. The remainder are small convenience samples, typically recruited from sympathetic groups and settings, that are (in my view and that of detailed reviews) [4, 7] worthless for the question of child outcomes. These studies do not meet minimal scientific standards and are biased toward benign findings [8]. Asking patrons of a local LGBT [Lesbian, Gay, Bisexual, Transgender] bookstore or gay friends network about child outcomes is like surveying a Bible study about religiosity: the rosy picture is misleading about the larger population. Excluding such nonrandom or biased samples, fewer than 10 of the 74 studies remain.

*The Problem of Special Pleading.* The validity of Frank's critique is undermined by selective application, known as special pleading. Regarding the sample, my study notes that “same-sex parents were identified using the procedure described by Wainright et al.” [9] Wainright's three studies used the same data and sample of same-sex parents as mine, selected in the same way. The difference is those studies conflated two groups that I believe should be examined separately: (1) children residing with two same-sex parents and (2) children residing with two opposite-sex parents, one of whom was in a same-sex relationship with a third person. Wainright mixed these about equally sized groups together, finding no statistically significant differences in child outcomes compared to opposite-sex families [9–11]. My study examined the first group separately, finding some disadvantages for children with two same-sex parents; otherwise, my study replicated Wainright's.

Since Wainright reported benign outcomes from Add Health (the National Longitudinal Study of Adolescent to Adult Health) 12 years ago, Frank has not, to my knowledge, ever critiqued her methods. He features her studies on his website as evidence of no disadvantages for same-sex parented children. Yet when I report adverse outcomes using the same data, sample methods, and a subset of the same sample, he says he is “appalled.” When Wainright refers to the adults in her sample as “same-sex parents” (though over half are opposite-sex couples) and their children as “raised by same-sex parents,” Frank makes no criticism. When I use the same language for some of the same cases, he critiques it as “inaccurate.” One of these two responses must be wrong. If Wainright's study was valid and credible when it reported no differences, why is it a flawed study when I, using the same evidence and method, report differences? Every one of Frank's criticisms of my sampling methods applies to Wainright's, so if it is untenable and misleading for me to report adverse outcomes by these methods, it is equally untenable and misleading for Wainright to report benign outcomes.

*Errors in Criticism.* In fact, both my own and Wainright's conclusions are well founded and Frank makes numerous errors in his criticisms. Examine the central statements to support his claim that my description of the children as “raised by” same-sex parents is “wholly untenable”:

*“All he knows about his dataset is that his subjects, who ranged in age from 12 to 18, spent some of their teenage years with a parent who at some point had a same-sex partner.”*

That is not true. Following Wainright, I selected “families in which parents reported being in a marriage or marriage-like relationship with a person of the same sex”; [9] thus these are current relationships and none of the children are only living with “a parent who at some point had a same-sex partner.” It is true for Wainright that most parents identified a same-sex partner other than the child's other parent—to that extent this criticism may apply to her studies—but I excluded those cases from my sample, so all children have two parents in a same-sex relationship with each other. This point also renders Frank's census speculations irrelevant.

*“Since we don't know if that partner was ever actually a parent, legally or otherwise, it is inaccurate to characterize such households as ‘same-sex parented' …”*

Again, that is not true. In addition to asking parents about their partners, Add Health asked children about their relationship with all the adults they lived with. The partner parents in my sample were thus all designated* by the children in their care* as a parent, as their “mother,” “step-mother,” “father,” “step-father,” or similar. Unlike my study, Wainright did not consult this variable to confirm partner parentage, so Frank's criticism may apply to her sample.

*“It is even more inaccurate to claim that those living in these households were ‘raised by' same-sex parents, since we know nothing about the youths' parentage before their teenage years.”*

Again that is untrue, though Frank raises an important point related to family stability. We know a lot about the youth's parentage before their teenage years from these data. At Wave I, Add Health obtained good measures of adolescents' tenure with (length of time in the care of) each parent, with additional retrospective questions on childhood family conditions at Wave III. On the broadest measure of tenure, that is, time in the care of the longer-tenured parent, average parental tenure was close to current age and did not differ between same-sex and opposite-sex parent families. (This is not true for the cases which Wainright includes but I exclude, so this criticism may apply to her sample.) There are good grounds to say the children were “raised by” these parents.

Neither I nor Wainright reported the results for the tenure variable, which was not used in our studies, but in response to Frank's criticism I do now in Table 1. For both groups of children the average time spent with the longer-tenured parent is about 14 years, only about 1.5 years below their average age, indicating that most children remained in the care of at least one of their parents since infancy and with no difference between same-sex and opposite-sex parents.

The table also addresses Frank's concern that not accounting for shorter stability may overstate child disadvantages with same-sex parents. The time children have been in the care of both current parents is indeed much shorter with same-sex than opposite-sex parents, but the effect of this difference is not what Frank speculates. The table compares age-adjusted odds ratios by family type, by length of tenure with both current parents, for the three primary affective outcomes I examined. Both-parent tenure (ranging from 0 to 20 years) is grouped into four five-year categories; the reference category is five years or fewer. For children with opposite-sex parents, longer tenure with the current parents is associated with lower odds of depression, anxiety, and thoughts of suicide; but the evidence does not support a similar conclusion for same-sex parents, as Frank assumes. For children with same-sex parents, odds of suicide ideation are unchanged by both-parent tenure and odds of depression and anxiety are, if anything,* higher* with longer tenure. (In a previous study I examined tenure and family transitions in these data in more detail, with similar, and statistically significant, results.) [12] Thus, contrary to what Frank alleges, the lack of control for stability or tenure does not overstate, and may* understate*, negative child outcomes with same-sex parents.

I think I have addressed enough errors in Frank's critique to establish that his criticisms of my study are unfounded and that my findings are well justified. However, I doubt this will be convincing to him or those sharing his perspective, because what appears to disturb them is not the study methods but the findings. I suspect no evidence will convince Frank that children with same-sex parents may face unique and heightened struggles and difficulties. It is right to be appalled at that thought, but the most useful response is to try to understand the problem better, so as to address the conditions or provide support necessary to ameliorate the problem, not deny the evidence.

## Figures and Tables

**Table 1:** Association of family tenure with child outcomes, by family type (same-sex parents versus opposite-sex parents) in Add Health Wave I.

	Same-sex parents	Opposite-sex parents	Test
Tenure with longer parent (mean)	14.2	13.8 (.68)	Difference of means (*t*)
Tenure with both parents (mean)	7.3	12.5 (.02)	Difference of means (*t*)
Outcomes by tenure (both parents)			
Depression (OR, sig.)	2.16 (.34)	.85 (.000)	OR ≠ 1 (*t*)
Suicide ideation (OR, sig.)	1.00 (.99)	.90 (.003)	OR ≠ 1 (*t*)
Anxiety (OR)	1.22 (.89)	.91 (.05)	OR ≠ 1 (*t*)
*N* (unweighted)	20	18,266	

Odds ratios report age-adjusted logistic regression estimates for 5-year categories of tenure with both parents.

Numbers in parentheses report *p* value for the referenced test.
